# A Gadolinium DO3A Amide *m*-Phenyl Boronic Acid MRI Probe for Targeted Imaging of Sialated Solid Tumors

**DOI:** 10.3390/biomedicines9101459

**Published:** 2021-10-13

**Authors:** Christu Rajan, Jaya Seema, Yu-Wen Chen, Tsai-Chen Chen, Ming-Huang Lin, Chia-Huei Lin, Dennis Wen-Han Hwang

**Affiliations:** 1 Institute of Biomedical Sciences, Academia Sinica, Taipei 115, Taiwan; chrischem25@gmail.com (C.R.); seema@ibms.sinica.edu.tw (J.S.); hazelnut.chen.scu@gmail.com (T.-C.C.); sam320@ibms.sinica.edu.tw (M.-H.L.); rukiya@ibms.sinica.edu.tw (C.-H.L.); 2 Biomedical Translation Research Center, Academia Sinica, Taipei 115, Taiwan; bcde23400@ibms.sinica.edu.tw

**Keywords:** MRI, contrast agent, CA, DOTA, PBA, SA

## Abstract

We developed a new probe, Gd-DO3A-Am-PBA, for imaging tumors. Our results showed active targeting of Gd-DO3A-Am-PBA to sialic acid (SA) moieties, with increased cellular labeling in vitro and enhanced tumor accumulation and retention in vivo, compared to the commercial Gadovist. The effectiveness of our newly synthesized probe lies in its adequate retention phase, which is expected to provide a suitable time window for tumor diagnosis and a faster renal clearance, which will reduce toxicity risks when translated to clinics. Hence, this study can be extended to other tumor types that express SA on their surface. Targeting and MR imaging of any type of tumors can also be achieved by conjugating the newly synthesized contrast agent with specific antibodies. This study thus opens new avenues for drug delivery and tumor diagnosis via imaging.

## 1. Introduction

Magnetic resonance (MR) spectroscopy/imaging has become one of the most successful analytical techniques for a diverse array of applications in chemistry, physics, biology, materials, and medicine. The aims of this study were to develop innovative bio-inspired materials and methods for sensing, and to significantly enhance the sensitivity of detection of biomarkers using MR spectroscopy and imaging, a fundamental challenge in modern MR. Sensitivity enhancement is particularly important for the early detection of diseases, such as cancers and dementia, using MR molecular imaging [1,2].

Gadolinium contrast agents that enhance the quality of magnetic resonance imaging (MRI) are extensively used in the diagnosis and treatment of tumors [3,4,5]. Although a great deal of research had been carried out over the past decade, it is very hard to distinguish between tumor regions and normal regions, since only a small amount of contrast agent is retained in the tumor site via the enhanced permeability retention effect. Studies have revealed that, after the administration of gadolinium-based contrast agents, patients with renal failure develop nephrogenic systemic fibrosis (NSF) [6]. Hence, the European Medicine Agency has recommended restrictions on the use of some intravenous linear MRI contrast agents in order to avoid the adverse health effects associated with the administration of gadolinium [7]. Therefore, the synthesis of macrocyclic chelator CAs with high stability, selectivity, and less toxicity has become a focus of research. Extensive research is being undertaken toward the development of novel gadolinium probes with selective targeting, high tumor retention, and rapid clearance from nontarget tissues [8].

Contrast agents are often conjugated to specific targeting agents to actively target the selective moieties that are expressed on the tumor surface. Although these kinds of strategies show promise, many questions, such as the long-term stability and in vivo integrity of the newly developed contrast agents, remain unanswered [9,10,11]. Despite the high targeting efficiency, rapid nonrenal clearable compounds can exhibit severe accumulation in the liver and spleen, raising concerns of long-term toxicity. Hence, elimination of contrast agents is the key factor to be considered. Contrast agents with rapid elimination are preferred for clinical use since their accumulation in other organs can be effectively minimized. 

Phenyl boronic acid (PBA), which selectively recognizes the overexpressed sialic acid (SA) on the surface of cancer cells, has attracted considerable attention for use in tumor diagnosis and targeted drug delivery [12,13,14,15,16,17]. PBA appears to be highly selective for SA under acidic conditions, with almost no interference from other common sugars present in biological samples, a phenomenon which forms the basis for the use of PBA as a tumor-targeting agent. Other features of PBA, such as its high selectivity, nontoxicity, and non-immunogenicity, make it attractive [18,19,20]. Murine B16-F10 cells and their xenografts were used in the study because of the aberrant expression of SA on its cell surface. Increased total serum sialic acid levels have proved to be a useful marker for the detection and progression of melanoma. Moreover, the co-relation between the enhanced expression of SA and its involvement in tumor metastasis and melanogenesis in melanoma cells were well documented. Elevated levels of SA in ovarian cancer, glioma, lung cancer, leukemia, colorectal cancer, and breast cancer were also detected; hence, the study can also be extended to other types of tumors [21,22].

A previous in vitro study demonstrated covalent binding of a PBA-amide-conjugated DTPA ligand to SA expressed on the surface of C6 glioma cells [23]. A DTPA-bisamide with an amide moiety bearing PBA for interaction with the diol groups in the side chain of sialic acid has also been reported [24]. In another study, PBA in the amide copolymer AAPBA-DMAm showed appreciably high binding with SA, binding with nearly 60% of the cells in 1 h [25]. NMR studies on the interaction between PBA and SA have confirmed the reversible covalent binding on the diol functions through the formation of five- and six-membered ring esters. The conditional stability constant of the PBA-Sia ester was 11.6 M^−1^ in homogeneous solution [26]. Several in vivo studies into Gd-DOTA-en-PBA probes and their dimeric forms exhibiting two-site binding occurring through PBA ester formation and an electrostatic interaction that lasted up to 24 h on the tumor site have also been reported [27,28].

In this paper, we report the synthesis and evaluation of a new MR contrast probe, Gd-DO3A-Am-PBA, in which the amide moiety of dodecane triacetic acid (DO3A) was conjugated to *m*-PBA. Gd-DO3A-Am-PBA was designed to identify and validate the in vivo SA-specific molecular targeting of PBA. If achieved, we aimed to further validate the synthetic strategies and make improvements in the in vivo efficiency of the probe for tumor targeting and therapy.

## 2. Experimental

### 2.1. Materials

All chemicals that were purchased from Sigma-Aldrich (St. Louis, MO, USA) and Alfa Aesar Co. (Thermo Fisher GmbH, Kandel, Germany) were used as received. Com-bi-Blocks were purchased from San Diego, USA. Ion exchange resin Dowex 50 WX8 hydrogen form, with 200–400 mesh was purchased from Sigma Aldrich (St. Louis, MO, USA). Solvents (ACS grade) and raw materials were used without further processing or purifica-tion. Reactions were monitored using TLC plates, and column chromatography was gen-erally performed on silica gel. Thin-layer chromatography (TLC) was carried out on silica plates and visualized using iodine, UV lamps at 254 nm, or staining with KMnO_4_, ninhydrin solution, as appropriate. ^1^H and ^13^C NMR were recorded on a Bruker AV500 and AV600 MHz spectrometer and the spectra were analyzed on TopSpin 3.6.1 software (Bruker, Germany). The chemical shifts (δ) are reported in ppm and coupling constants (J) in Hz relative to TMS (0.00) Mass spectra were obtained on the Bruker BIO-TOF III. HPLC (Shimadzu, Kyoto, Japan) was used for purity calculation.

### 2.2. Chemistry

Detailed *information* about the *synthesis* and characterization of Gd-DO3A-Am-PBA are *included* in the Supplementary Materials. All compounds were confirmed using 1HNMR, 13CNMR, and mass spectra. The purity of the contrast agent was found to be 97.7% in HPLC. The amount of Gd^3+^ in Gd-DO3A-Am-PBA was quantified by inductively coupled plasma atomic emission spectroscopy (ICP-MS). Frequent analysis of Gd-DO3A-Am-PBA by NMRD and ICP-MS confirmed the long-term stability of the contrast agent.

### 2.3. Cell Culture and Animals

B16-F10 melanogenic cells were cultured in Dulbecco’s Modified Eagles Medium (DMEM, Gibco, NY, USA) supplemented with 10% heat-inactivated fetal bovine serum (FBS, Gibco) and 100 U/mL of penicillin/streptomycin (Gibco). Cells were maintained in a humidified incubator at 37 °C under 5% CO_2_. Non-melanogenic cells were obtained by growing B16-F10 melanogenic cells in RPMI (Hyclone) medium supplemented with 10% heat-inactivated FBS and 100 U/mL of penicillin/streptomycin. The cells were incubated at 37 °C in a humidified atmosphere of 10% CO_2_.

Nude mice were purchased from BioLASCO Co., Ltd. (Taipei, Taiwan) and maintained in a specific-pathogen-free vivarium with a well-controlled environment with a 12-h/12-h light/dark cycle and controlled humidity and temperature. Female mice 8–10 weeks old, weighing approximately 22–25 g were used for all in vivo experiments. All experimental procedures were approved by the Institute of Animal Care and Utilization Committee at Academia Sinica, Taipei, Taiwan. For tumor induction, 1 × 10^6^ melanoma cells were suspended in 100 μL of PBS and injected subcutaneously in the right flank of nude mice. Tumor-bearing mice were randomly divided into two groups (*n* = 6 for each group) for intratumor and intravenous injections.

### 2.4. Relaxivity Measurement

For phantom relaxivity studies, Gd-DO3A-Am-PBA and Gadovist with three gadolinium concentrations (0.125, 0.25, and 0.5 mM) were prepared by diluting the samples in pure water. The test tubes were fixed in a polystyrene holder and then placed inside the head coil. After a three-plane localizer scan, the phantom was scanned on a 7T MRI scanner (PharmaScan 70/16, Bruker, Germany) by a series of pulse sequences (parameters are given in the Supplementary Materials). The *T*_1_ and *T*_2_ values of the phantom were evaluated, and the relaxation rates, *R*_1_(=1/*T*_1_) and *R*_2_(=1/*T*_2_), were obtained from the slopes of linear fits of the experimental data.

### 2.5. NMRD Measurements

Nuclear magnetic relaxation dispersion (NMRD) profiles for 2 μmol of Gd-DO3A-Am-PBA and Gadovist were acquired on a SpinMaster FFC-2000 (Stelar s.l.r., Mede (PV), Italy) fast field cycling NMR relaxometer over a magnetic field strength ranging from 0.00024 to 0.94 T, corresponding to a proton Larmor frequency range of 0.01–40 MHz. Measurements were performed on 200-μL samples contained in 5-mm-diameter, 177.8-mm-long NMR tubes. The temperature was controlled with a Stelar VTC91 airflow heater equipped with a calibrated copper-constantan thermocouple. The stability of Gd-DO3A-Am-PBA was also investigated by obtaining NMRD profiles for freshly prepared solutions and those stored for up to six months at room temperature. All NMRD measurements were recorded at a temperature of 25 °C.

### 2.6. Cytotoxicity Studies

3-(4,5-Dimethylthiazol-2-yl)-2,5-diphenyltetrazolium bromide (MTT) assay kits (Roche, Mannheim, Germany) were used to determine the cell toxicity of Gd-DO3A-PBA, Gadovist, and GdCl_3_ to B16-F10 melanoma cells. Cells were seeded in 100 μL of medium in a 96-well, flat-bottomed plates and incubated under 5% CO_2_ at 37 °C for 24 h. Solutions of Gd-DOTA-Am-PBA, Gadovist, and GdCl_3_ were added to the cells to obtain Gadolinium concentrations ranging from 25 to 5000 μM. Following incubation for 24 h, 10 μL of MTT reagent was added to each well and incubated for an additional 4 h in a CO_2_ incubator. The purple formazan products formed were dissolved by adding 100 μL of the solubilization buffer supplied with the MTT assay kits. The plate was incubated overnight at room temperature, and the absorbance was measured on a microtiter *plate reader* (*SpectraMax 190*; Molecular Devices, San Jose, CA, USA) at 570 nm (*A*_570_) using 750 nm (*A*_750_) as background absorbance. Untreated cells were assayed similarly, to obtain the value of the control as 100% viability. The cell viability was calculated using the equation below. All assays were performed in triplicate.


$$\%\;\mathrm{Cell}\;\mathrm{viability}=[\frac{(A_{570}-A_{750})\mathrm{treated}\;\mathrm{sample}}{(A_{570}-A_{750})\mathrm{control}\;\mathrm{sample}}]\times 100$$

### 2.7. In Vitro Studies

Cell labeling studies were performed to determine the ability of Gd-DO3A-Am-PBA to label melanogenic cells that express SA on their surface. Non-melanoma cells and Gadovist were used as cell and contrast agent references, respectively. Melanoma and non-melanoma cells were grown to 60% confluence in six-well cell culture plates. The medium was replaced with fresh medium containing 0.5 mM of Gd-DO3A-Am-PBA. After one hour, the medium was removed, and the cells were washed three times with PBS to remove unbound Gd-DO3A-Am-PBA. The Gd^3+^ content of the cells was analyzed using ICP-MS, after nitric acid digestion (Sigma Aldrich) in a water bath at 80 °C for at least 8 h. For time-dependent cellular uptake studies, melanoma cells were treated with 0.5 mM of Gd-DO3A-Am-PBA and Gadovist, harvested at different time intervals (2 h, 4 h, and 24 h), and the amount of Gd^3+^ was quantified as previously described. An Olympus inverted microscope (Olympus Corporation, Tokyo, Japan) was used to qualitatively investigate the cell growth, morphological alterations, and viability of the cells before and after each labeling.

### 2.8. In Vivo MRI

To validate the MR performance of Gd-DO3A-PBA in vivo, two cohorts of female nude mice (*n* = 6 for each experiment) were subcutaneously injected with 1 × 10^6^ B16-F10 melanoma cells in the right flank. After 8 to 10 days, the first group of mice was intravenously administered 0.1 mmol/kg of Gd-DO3A-Am-PBA in the lateral tail vein. The second group of mice received the same dose of Gadovist as control. Fat suppressed T_1_-weighted multislice multiecho MR coronal images were recorded before and after 10 min, 30 min, 50 min, 70 min, 90 min, 110 min, 130 min, 4 h, or 24 h of contrast agent administration. For intratumor injections, 0.1 μmol/kg of Gd-DO3A-Am-PBA and Gd-DOTA were injected. All images were acquired on a 7T PharmaScan 70/16 MR scanner (Bruker, Germany). The mice were initially anesthetized with 5% isoflurane at 1 L/min air flow. When fully anesthetized, each animal was placed in a prone position and fitted with a custom-designed head holder inside the magnet. Isoflurane was then maintained at 1–1.2%, with a 1 L/min air flow throughout the experiments. Images were acquired using 38-mm quadrature coils as both transmitter and receiver. The MRI parameters are detailed in the Supplementary Materials.

### 2.9. Hematoxylin and Eosin Staining

All mice were perfused immediately after the last scan. The tumor, heart, liver, and kidney were removed, washed with PBS, fixed in 4% paraformaldehyde, dehydrated in a graded series of ethanol, and embedded in paraffin wax using a tissue embedding machine. Sections 5 mm in thickness were prepared and stained with hematoxylin and eosin. The tissue morphology was observed under a microscope. Histologic slices were scanned in a Pannoramic 250 FLASH II scanner and processed using CaseViewer 2.0 software (3D-HISTECH, Budapest, Hungary).

## 3. Results and Discussion

### 3.1. Sythesis of Gd-DO3A-Am-PBA

**Figure 1:**
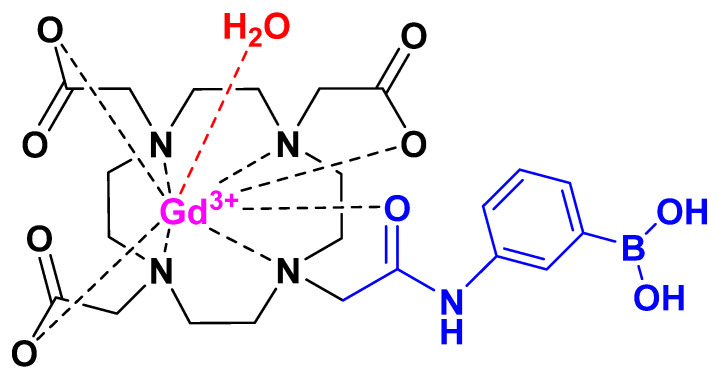
Molecular structure of Gd-DO3A-Am-PBA.

Various attempts have been made to develop contrast agents with higher CA loading, better relaxivity and targetability, improved contrast, and faster clearance and stability. Our new probe, Gd-DO3A-Am-PBA (Figure 1), which was prepared in a straightforward manner modified from previous successful procedures, was intended to target SA, overexpressed on certain solid tumors with high specificity, acceptable retention phase, and rapid renal clearance.

**Scheme 1:**
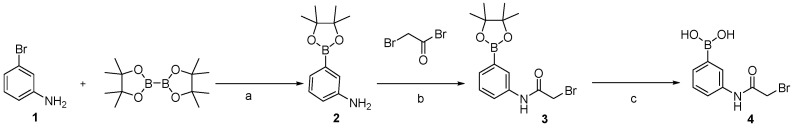
Synthesis of boronic acid (BA)-linker: (a) Pd(dppf)Cl_2_, CH_3_COOK, dioxane, 80 °C, 12 h, 91%; (b) K_2_CO_3_, CH_2_Cl_2_, 0 °C to rt, 5 h, 80%; (c) NaIO_4_, 2N HCl, THF: H2O (4:1), rt, 5 h, 85%.

**Scheme 2:**
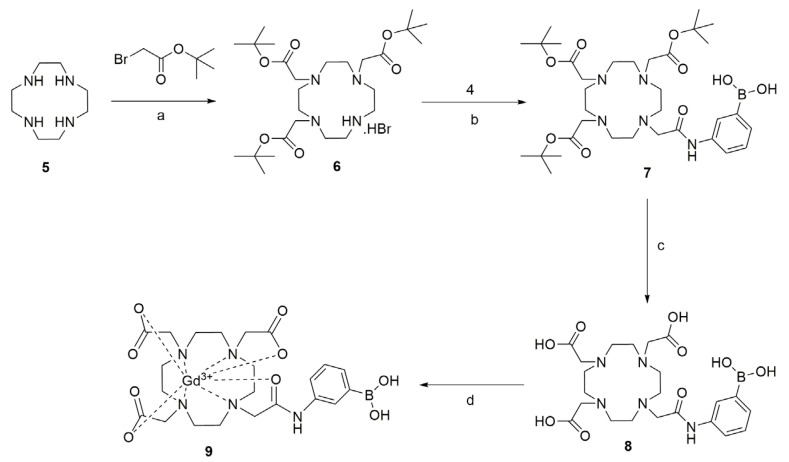
Synthesis of Gd-DO3A-Am-PBA: (a) K2CO_3_, CH_3_CN, 0 to 10 °C 72 h, 60%; (b) K2CO_3_, CH_3_CN:C_2_H_5_OH (2:1), rt, 24 h, 50%; (c) TFA:CH_2_Cl_2_ (1:1), rt, (d) GdCl_3_.6H_2_O, H_2_O, pH 5–6, 50 °C, 12 h.

Gd-DO3A-Am-PBA was synthesized as shown in Scheme 1 and Scheme 2. The synthesis of boronic acid linker (4) (Scheme 1) begins with 3-bromo aniline, which was converted to 3-amino phenyl boronic ester (2) by Pd(dppf)Cl_2_ and dioxoborolone ester bis(pinacolato)diborane [29]. The reaction of (2) with bromoacetyl bromide produced bromo acetamido phenyl boronic ester (compound 3) [30]. This compound was further treated with NaIO_4_ in acidic medium to obtain (3-(2-bromoacetamido) phenyl) boronic acid (4).

The synthesis of Gd-DO3A-Am-PBA is shown in Scheme 2. Tri tertiary butyl cyclene hydro bromide salt (6) was synthesized according to a published procedure [31]. Cyclene was treated with tertiary butyl bromoacetate and sodium acetate trihydrate in DMAc solvent at 0 to 8 °C for 60 h to produce (6), which was further reacted with (4), potassium carbonate in a mixture of solvents ethanol and acetonitrile at room temperature for 24 h to obtain (7). Deprotection of (7) was carried out by TFA/DCM (1:1) at room temperature to obtain the ligand (8) [27]. It was further purified using ion exchange resin. Finally, the complexation of (8) with GdCl_3_.6H_2_O yielded Gd-DO3A-Am-PBA (9) as a white solid (See Supplementary Materials for ^1^H and ^13^C NMR spectral data). Relaxivity properties, in vitro cell binding, in vivo tumor targeting, retention time, and renal clearance of Gd-DO3A-PBA were accessed and compared to those of Gadovist.

### 3.2. Measurements of Relaxation Rate

**Figure 2:**
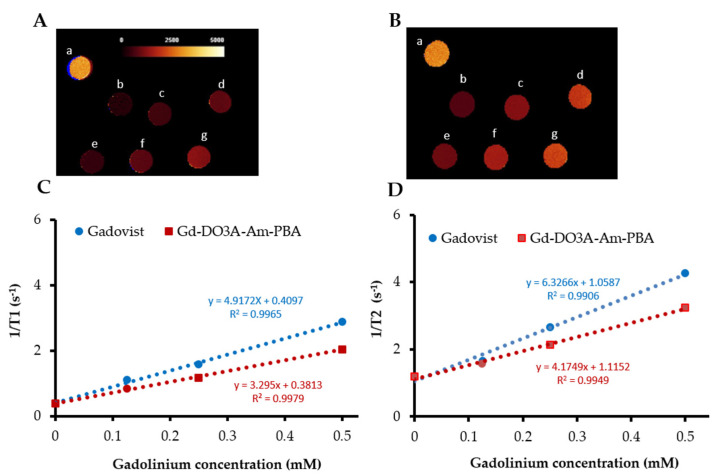
(**A**) Spin-echo (SE) *T*_1_-weighted MR images of the phantoms corresponding to the concentrations 0.125, 0.25, and 0.5 mM for water (a), Gd-DO3A-Am-PBA (b, c, d) and Gadovist (e, f, g). (**B**) Spin-echo (SE) *T*_2_-weighted MR images of phantoms at the same concentration for water (a), Gd-DO3A-Am-PBA (b, c, d), and Gadovist (e, f, g). All measurements were performed in deionized water, pH 7, using 7T MRI scanner at room temperature. (**C**) Longitudinal relaxation rate (*R*_1_) of Gd-DO3A-Am-PBA (red) and Gadovist (blue). (**D**) Transverse relaxation rate (*R*_2_) of Gd-DO3A-Am-PBA (red) and Gadovist (blue). Relaxivity values *R*_1_ or *R*_2_ were obtained from the slopes of linear fits of the experimental data.

To investigate the potential use of Gd-DO3A-Am-PBA in MRI, the paramagnetic properties of the compound were studied using a 7T MRI scanner, and the results were compared with those of Gadovist. Phantom contrast images of 1.5 mm thickness were obtained perpendicular to the scan plane of the tubes for 0.125 mM, 0.25 mM, and 0.5 mM concentrations of Gd-DO3A-Am-PBA and Gadovist. All of the concentrations were easily visualized from the phantom images (Figure 2A,B). Comparative analysis of the results indicated that Gd-Gd-DO3A-Am-PBA can enhance the contrast as well as Gadovist. *R*_1_ and *R*_2_, the relaxation rates of the phantoms, are given in Figure 2C,D, respectively. The *R*_1_ and *R*_2_ of Gd-DO3A-Am-PBA and Gadovist were calculated to be 3.3041 mM^−1^s^−1^ and 4.4546 mM^−1^s^−1^, and 4.8125 mM^−1^s^−1^ and 6.8311 mM^−1^s^−1^, respectively (Table 1). The *R*_2_/*R*_1_ ratio of Gd-DO3A-Am-PBA (1.3655) is similar to that of Gadovist (1.45755). Usually, *T*_1_ contrast agents have lower *R*_2_/*R*_1_ ratios (<5), while *T*_2_ contrast agents have higher *R*_2_/*R*_1_ ratios (>10). The in vitro relaxivity properties obtained highlight the possibility of using GD-DO3A-Am-PBA as a potential *T_1_*-*weighted MRI contrast agent* [32].

### 3.3. Measurement of Relaxivity and Stability

**Figure 3:**
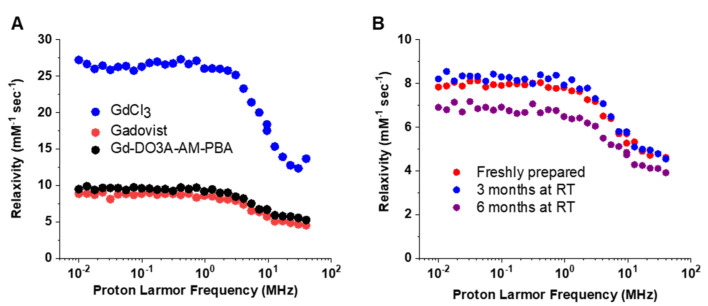
(**A**) Measured 1/*T*_1_ NMRD profiles of Gd-DO3A-Am-PBA (black), Gadovist (red), and GdCl_3_ (blue) with 2 µmol of gadolinium. (**B**) Measured 1/T_1_ NMRD profiles of freshly prepared (red) and three months (blue) and six months (purple) room temperature stored Gd-DO3A-Am-PBA. All measurements were made in pure water, pH 7, at 25 °C.

^1^H NMRD profiles of Gd-DO3A-Am-PBA, Gadovist, and GdCl_3_ were recorded for comparison, and to study the field-dependent relaxivity. The black, red, and blue dots represent the relaxivity of Gd-DO3A-Am-PBA, Gadovist, and GdCl_3_, respectively (Figure 3A). The relaxivity values obtained indicate that Gd-DO3A-Am-PBA is as effective as Gadovist. Safety is another important parameter that has to be considered when designing and synthesizing MRI contrast agents for clinical applications. Recent in vivo research findings have emphasized the importance of evaluating the contrast agents for stability in order to minimize gadolinium dissociation from the chelating agent during storage to decrease toxicity and reduce inaccuracy of the outcomes of in vivo experiments [33]. The stability of Gd-DO3A-Am-PBA was investigated by acquiring the NMRD profiles of the freshly prepared solutions, those stored at 4 °C (data not shown), and solutions stored at room temperature for least six months. As shown in Figure 3B, curves acquired for freshly prepared Gd-DO3A-Am-PBA and that stored at room temperature for up to six months are almost constant. The comparative results and the reproducibility of relaxivities obtained for Gd-DO3A-Am-PBA stored at 4 °C and room temperature indicated that Gd-DO3A-Am-PBA had good stability up to 3 months.

**Figure 4:**
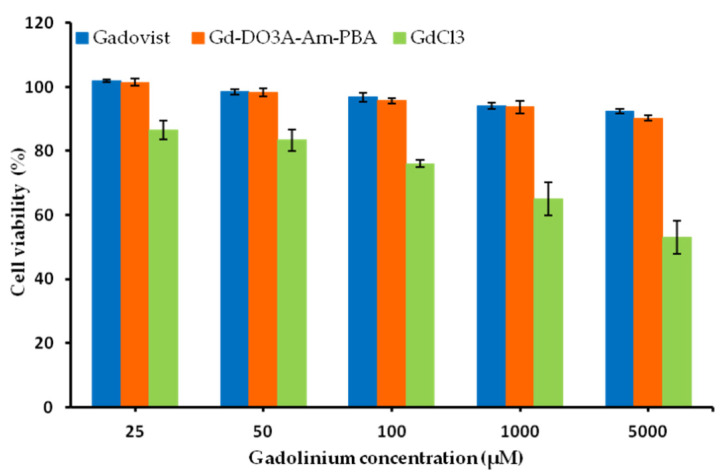
MTT assays. Cell viability (%) of melanoma cells exposed to varying concentrations of Gd-DO3A-Am-PBA, Gadovist, and GdCl_3_ for 24 h. Error bars represent mean ± standard error values from three independent experiments.

We investigated the dose-dependent viability of melanoma cells treated with Gd-Gd-DO3A-Am-PBA, Gadovist, and GdCl_3_ at concentrations ranging from 0 to 5 mM, using MTT assays. Neither Gd-DO3A-Am-PBA nor Gadovist, which was used as a control, affected the viability of the cells, and therefore appeared to be nontoxic (Figure 4). GdCl_3_ showed concentration-dependent cytotoxicity. Obvious differences between the toxic potency of GdCl_3_ and the other two contrast agents were detected, even at the lowest concentration tested. The toxicity exhibited by GdCl_3_ might be due to the fast internalization of Gd^3+^ by cells. As a rule of thumb, the chelator should bind tightly to Gd^3+^ ions in order to prevent its release into the circulation. The high viability of cells treated with Gd-DO3A-Am-PBA and Gadovist illustrated that Gd^3+^ ions in Gd-DO3A-Am-PBA are tightly bound, making the complex more stable, and thereby preventing its release into the medium. The high cell survival and negligible toxicity observed suggest that our newly synthesized Gd-DO3A-Am-PBA is safe for use as an MRI probe.

### 3.4. Binding Efficiency

**Figure 5:**
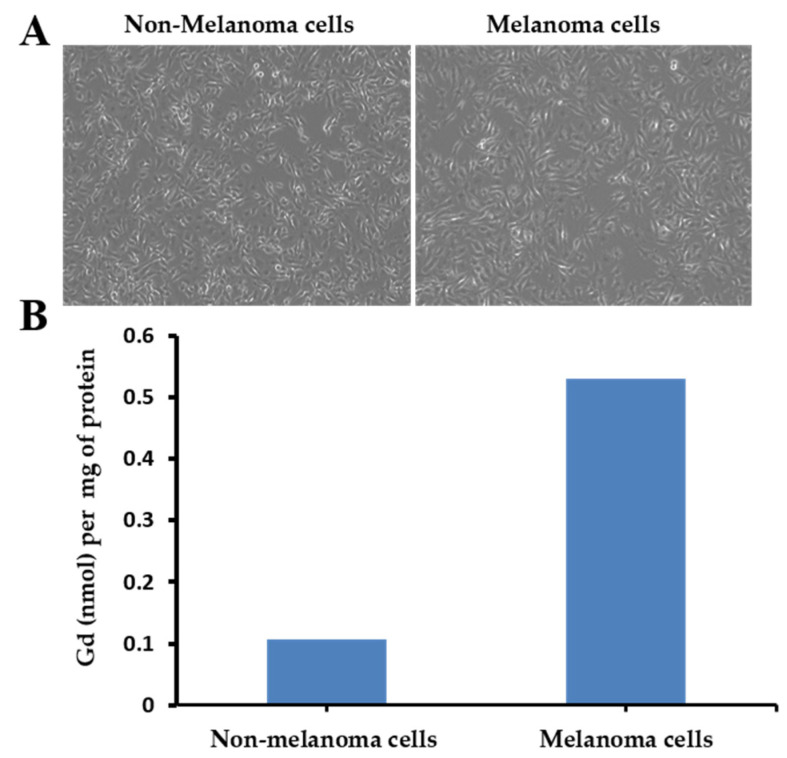
In vitro cell labeling. (**A**) Phase-contrast micrographs of non-melanoma and melanoma cells one hour after the addition of 0.5 mM Gd-DO3A-Am-PBA (10× magnification). (**B**) Quantification of binding affinity by ICP-MS. Results are expressed as per mg of protein.

**Figure 6:**
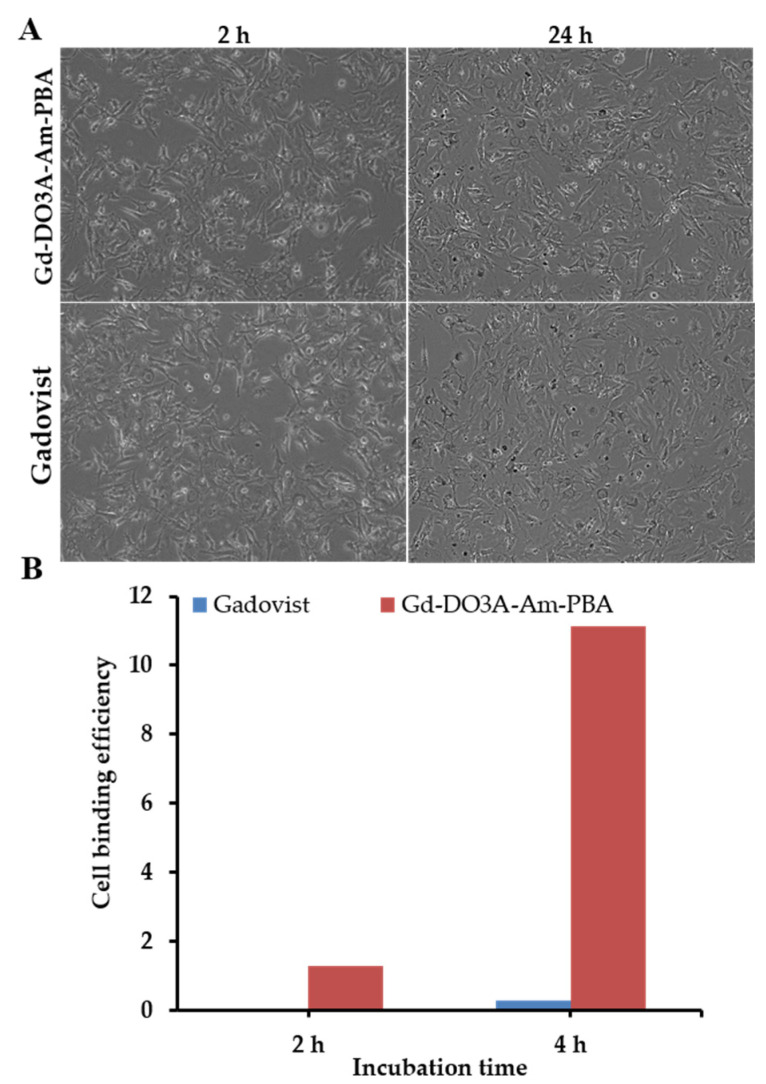
Effect of incubation time on cell labeling. (**A**) Phase-contrast micrographs of melanoma cells 2 h and 24 h after the addition of 0.5 mM Gd-DO3A-Am-PBA or Gadovist (10× magnification). (**B**) Comparison of binding efficiency of Gd-DO3A-Am-PBA or Gadovist after 2 h and 4 h of cell incubation.

The in vitro binding affinity of Gd-DO3A-Am-PBA to SA was investigated using melanoma cells, with non-melanoma cells as the control group [34]. For in vitro cell labeling, the two groups of cells were treated with 0.5 mM of Gd-DO3A-Am-PBA for 1 h at 37 °C. Then, the cellular uptake was assessed by measuring the internalized Gd^3+^ by ICP-MS of washed cell pellets. One hour after labeling, both melanoma and non-melanoma cells under a phase-contrast microscope exhibited characteristic spindle-like morphology, homogeneous distribution, and no morphological abnormalities, shrinking, or rounding of cells (Figure 5A). ICP-MS results showed higher binding of Gd-DO3A-Am-PBA by melanoma cells than by non-melanoma cells (Figure 5B). This marked Gd-DO3A-Am-PBA affinity of melanoma cells is directly proportional to the amount of SA on the cell surface. It was clear that Gd-DO3A-Am-PBA specifically targeted SA and was tumor-specific, as it labeled melanoma cells more heavily than non-melanoma cells. Further in vitro experiments that utilized ICP-MS to evaluate the effect of incubation time (2 h, 4 h, and 24 h) on cell labeling were performed. No defects in cell morphology and no detached cells were detected 24 h after the addition of Gd-DO3A-PBA or Gadovist (Figure 6A). At 2 h, Gd-DO3A-Am-PBA depicted around 29-fold higher binding toward the melanoma cells (Figure 6B). The significantly higher affinity depicted by Gd-DO3A-Am-PBA (Figure 6B) clearly specify the defined specificity of the same toward SA. In contrast, little binding was displayed by the nonspecific agent, Gadovist, that served as the control. This defined variation in the cell targeting efficiency between the two contrast agents clearly stipulate the binding interaction between Gd-DO3A-Am-PBA and cell surface-expressed SA. From the in vitro results, it is evident that Gd-DO3A-Am-PBA can be efficiently used for tracking and visualizing tumors that express characteristic surface marker SA. The in vitro results obtained also demonstrate the potential to use this approach in in vivo studies.

### 3.5. In Vivo MRI of Tumor Model Mice

**Figure 7:**
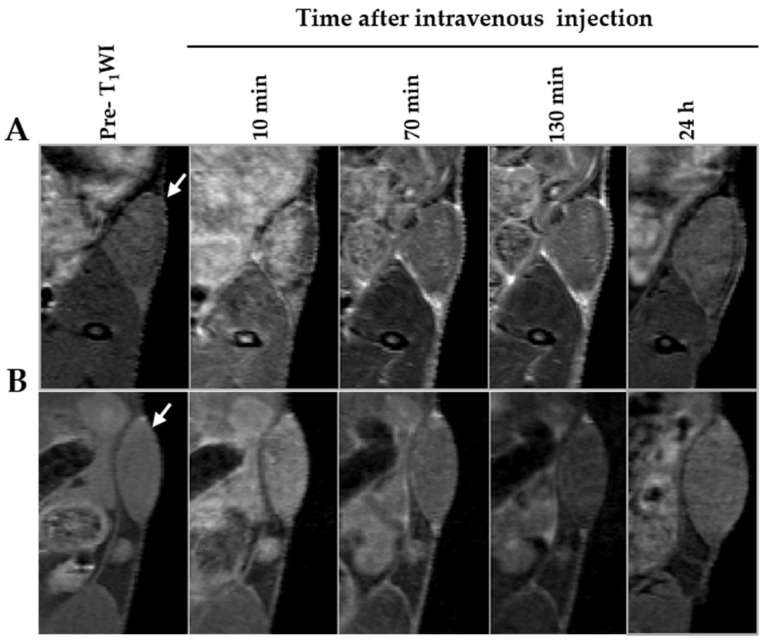
In vivo magnetic resonance imaging (MRI). T_1_-weighted MR images of B16-F10 melanoma tumor-bearing mice after the intravenous administration of Gd-DO3A-Am-PBA (**A**) or Gadovist (**B**) at different time points (pre-and post-injection of contrast agent at 10 min, 70 min, 130 min, and 1440 min) with 0.1 mmol/kg of gadolinium. The pre-contrast T_1_ shown were acquired immediately prior to injection (0 min). Tumors are indicated by arrows.

**Figure 8:**
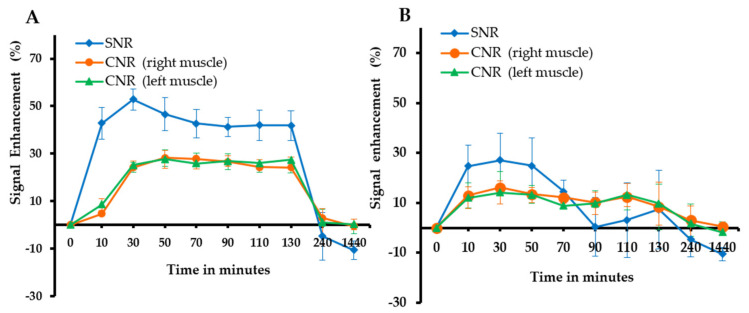
Quantification and comparison of the SNR and CNR in the tumor region measured after the intravenous injection of Gd-DO3A-Am-PBA (**A**) or Gadovist (**B**). Error bars represent mean ± standard error values. SNR: signal-to-noise-ratio; CNR: contrast-to-noise ratio.

The performance of Gd-DO3A-Am-PBA was further evaluated by intravenously injecting 0.1 mmol/kg of Gd-DO3A-Am-PBA via the tail vein. The changes in signal intensity were compared with those induced by Gadovist. T1-weighted spin echo MR images were acquired before and 10 min, 30 min, 70 min, 110 min, 130 min, 240 min, and 1440 min after injection (Figure 7A,B). The acquired data were analyzed using MR Vision software (version 1.6.6, MRVision, Winchester, MA, USA). Regions of interest (ROIs) were drawn for the tumor, and the signal-to-noise ratio (SNR) was calculated. The SNRs of images acquired at different time points were normalized with the background signal, and expressed as percentage changes relative to their respective baseline values. In order to confirm that the enhanced retention of Gd-DO3A-Am-PBA was tumor-specific, the contrast-to-noise ratios (CNRs) between neighboring muscle tissues and tumors were also assessed. Following intravenous injection, Gd-DO3A-Am-PBA exhibited around 6% contrast enhancement at 10 min, with a maximum signal enhancement of 24% at 30 min post injection, and a continuous retention phase that extended up to 2 h (25%) (Figure 8A). After 2 h, the signal intensity began to drop, with a complete washout at 4 h. On the other hand, with Gadovist, the signal enhancement detected in the tumor region persistently increased, with a peak at 10 min (12%), followed by a fast decay as early as one hour (Figure 8B). The fast washout of Gadovist from the tumor region might be due to the non-targeted binding of Gadovist to melanoma tumors or the passive accumulation via the tumor vascular permeability. Conversely, efficient accumulation of Gd-DO3A-Am-PBA in the tumor region was due to the specific binding of PBA ligand to the hypersialated regions in melanoma tumors. The targeted binding of our probe, Gd-DO3A-Am-PBA, to SA at earlier time points was supported by prior studies, according to which the PBA in the amide copolymer, AAPBA-DMAm, showed appreciably high binding toward SA. In this in vitro study, cells with SA could bind to AAPBA-DMAm within 60 min. This study strongly supports the binding of our new probe at earlier time points [25]. In previous studies, DOTA-en-PBA and their dimeric analogs were synthesized, and their binding efficiency was assessed in vivo at 240 min and 1440 min using MRI [27,28]. All the above probes were enhanced in tumors at 240 min and 1440 min. The difference in the tumor targeting and contrast agent washout observed between DOTA-en-PBA or their dimeric forms and our Gd-DO3A-Am-PBA might be attributed to two main causes. First, in Gd-DO3A-Am-PBA, the 3-boronic acid was conjugated to the ligand by amide bonds (-NH-CO); hence, binding occurs via ester formation between the vicinal diol functions of SA and the hydroxy groups of PBA. In Gd-DOTA-EN-PBA, PBA was conjugated to DOTA via an ethylenediamine (en) spacer (-NH-CH2-), which facilitates covalent binding of SA by the PBA moiety and the electrostatic interaction between the positively charged ammonium group and the negatively charged cell surface. Secondly, the reversible molecular recognition chemistry between the PBA and SA explains the shorter retention time and rapid washout of Gd-DO3A-Am-PBA. Furthermore, the mechanism behind the tumor targeting of Gd-DO3A-Am-PBA can also be proven and well explained from a previous preliminary in vitro study conducted by Kristina Djanashvili et al. Based on this study, 4 h exposure of Tb–DTPA, without PBA, did not depict any interaction with the cells, while Tb–DTPA–(EN)2 showed a mild increase in cell binding due to the electrostatic interaction of the complex toward the negatively charged cell surface. Interestingly, higher levels of activity were observed after incubating with Tb–DTPA–(PBA)2 due to the covalent binding of PBA with SA [23].

In addition, we also investigated the in vivo targeting and binding efficiency of Gd-DO3A-Am-PBA intratumorally. For this study, 0.1 μmol/kg of the contrast agents were injected into mice grafted with melanoma tumors. T1-weighted spin echo MR images were acquired before and 10 min, 1 h, 2 h, 4 h, and 24 h after injection (data not shown). Gd-DO3A-Am-PBA accumulated and was rapidly distributed at the tumor region, presenting a high intensity until 2 h after injection. This observation confirmed that Gd-DO3A-Am-PBA has higher binding affinity, compared to Gadovist due to the binding of BA to SA, and thus generate a local high concentration of Gd-DO3A-Am-PBA (Figure S2).

**Figure 9:**
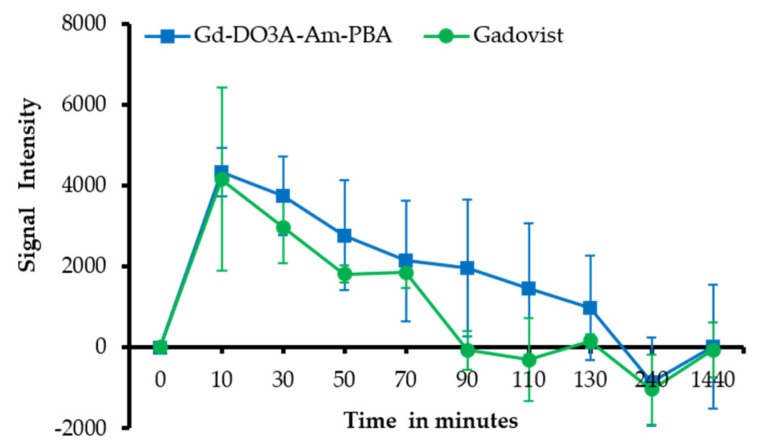
Quantification of kidney SNR after the intravenous injection of Gd-DO3A-Am-PBA and Gadovist. Error bar represent mean ± standard error values from three independent experiments. SNR: signal-to-noise-ratio.

Gd-DO3A-Am-PBA exhibited a higher washout rate from muscle and a lower washout rate from tumor, whereas Gadovist showed similar washout from both muscle and tumor sites. This trend confirmed the specific and targeted accumulation of Gd-DO3A-Am-PBA in tumors, which in turn supports the contention that it can be a potential contrast *agent* probe for MR of tumors. In addition, with normally functioning kidneys, most of the administered GBCAs, regardless of which agent was given, should be eliminated in <120 min after injection, and >95% by 24 h. For safety reasons, rapid clearance is one of the key requirements for MRI contrast agents. This is necessary to prevent chronic toxicity due to the slow deposition of dissociated free metal ions in specific tissues or organs. The corresponding signal intensities of kidneys (Figure 9) revealed that Gd-DO3A-Am-PBA enters into the washout phase and is excreted as early as 4 h through renal filtration, which is a positive sign.

Finally, it is well known that sensitivity, specificity, and safety are the three strategies to consider while designing a successful contrast agent. Our probe, Gd-DO3A-Am-PBA, was designed to meet all the above criteria so that it could facilitate rapid binding, quick MR imaging, and efficient elimination from the body. As expected, Gd-DO3A-Am-PBA exhibited maximum targetability as early as 30 min, with a retention time of 2 h and renal elimination within 4 h. We assume that a 2-h retention time is more than sufficient for tumor detection and imaging. Longer retention of contrast agents may evoke potential toxicity, which is a major hurdle in the clinical translation of contrast agents. Hence, herein, we propose a novel probe, Gd-DOTA-Am-PBA, with “faster binding and rapid clearance” and believe that our detailed study on this probe would help not only researchers but also physicians who use contrast agents on a daily basis for the accurate diagnosis of tumors.

Altogether, the persistent contrast enhancement at 30 min, plateau signal intensity for 120 min, and total washout within 4 h exhibited by our newly synthesized amide probe, Gd-DO3A-Am-PBA, could make it a probe of choice for the safe and early diagnosis and treatment of tumors.

### 3.6. Histological Analysis

**Figure 10:**
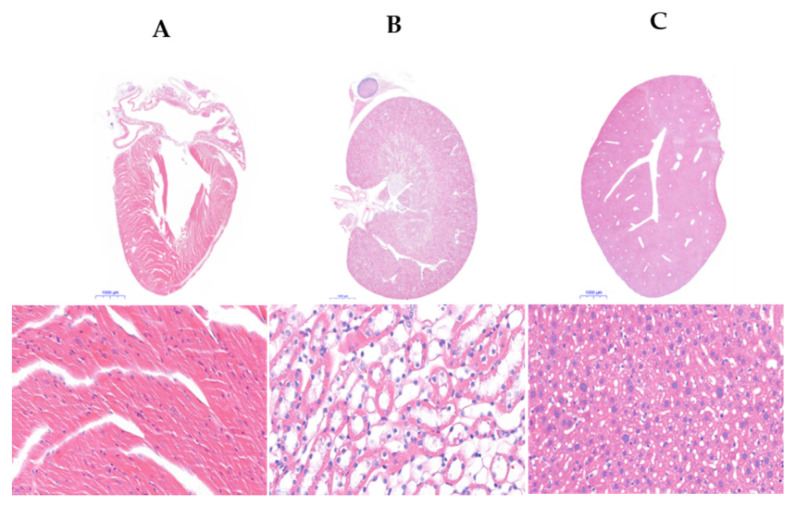
Histological specimens of heart (**A**), kidney (**B**), and liver (**C**) samples of mice injected with Gd-DO3A-Am-PBA. Mice were sacrificed immediately after 24-h MR imaging. All organs presented a normal appearance in macroscopic and microscopic observations. The scale bar is 25 μm.

MRI was followed by histological analysis of vital organs heart, liver, and kidney to observe the local and systemic toxicity induced by Gd-DO3A-Am-PBA. No alterations in the cellular integrity and tissue morphology were detected in those vital organs after the histological examination of the slides (Figure 10). All the internal organs presented normal macroscopic and microscopic profiles without any signs of necrosis, inflammation, or pigmentation. Altogether, this study demonstrates suitable tolerance and safety of Gd-DO3A-Am-PBA for detecting SA expressing tumors.

## 4. Conclusions

In this study, we compared and evaluated in depth the magnetic relaxivity, tumor specificity, retention time, and renal clearance of the newly synthesized probe Gd-DO3A-Am-PBA to those of Gadovist. The probe Gd-DO3A-Am-PBA displayed similar relaxivities to and greater tumor retention than Gadovist. The pronounced rapid and high retention of the contrast agent (CA) observed at the tumor site with relatively faster renal clearance suggest the possibility of using this probe for tumor diagnosis and therapy. Overall, the results of the in vitro experiments, together with the in vivo studies, suggest that Gd-DO3A-Am-PBA can be added to the growing list of contrast agents.

## Figures and Tables

**Table 1:** Calculated longitudinal relaxivity *R*_1_, *R*_2_, and the relaxation rate ratio *R*_2_/*R*_1_ for Gd-DO3A-Am-PBA and Gadovist at room temperature using 7T MRI scanner.

(mM^−1^sec^−1^)	*R*_1_	*R*_2_	*R*_1_/*R*_2_
Gd-DO3A-Am-PBA	3.295	4.1749	1.2670
Gadovist	4.9172	6.3266	1.2866

## Supplementary Materials

The following are available online at www.mdpi.com/article/10.3390/biomedicines9101459/s1. Figure S1: 40x brightfield images of non-melanoma and melanoma cells under light microscope, Figure S2: Quantification and comparison of SNR in the tumor region measured after the intratumor injection of Gd-DOTA and Gd-DO3A–Am-PBA.
