# Supplementary material for: A Gadolinium DO3A Amide m-Phenyl Boronic Acid MRI Probe for Targeted Imaging of Sialated Solid Tumors
Source: Biomedicines. 2021 Oct 13;9(10):1459. doi: 10.3390/biomedicines9101459 (PMC8533322; doi:10.3390/biomedicines9101459)
Supplement: Supplementary file 1 [file biomedicines-09-01459-s001.zip › biomedicines-1410622-supplementary.pdf]

biomedicines

Supporting Information

# A Gadolinium DO3A Amide *m*-Phenyl Boronic Acid MRI Probe for Targeted Imaging of Sialated Solid Tumors

**Christu Rajan [1,†], Jaya Seema [1,†], Yu-Wen Chen [2], Tsai-Chen Chen [1], Ming-Huang Lin [1], Chia-Huei Lin [1] and Dennis Wen-Han Hwang [1,2,*]**

[1] Institute of Biomedical Sciences, Academia Sinica, Taipei 115, Taiwan; chrischem25@gmail.com (C.R.); seema@ibms.sinica.edu.tw (J.S.); hazelnut.chen.scu@gmail.com (T.-C.C.); sam320@ibms.sinica.edu.tw (M.-H.L.); rukiya@ibms.sinica.edu.tw (C.-H.L.)
[2] Biomedical Translation Research Center, Academia Sinica, Taipei 115, Taiwan; bcde23400@ibms.sinica.edu.tw
* Correspondence: dwhwang@ibms.sinica.edu.tw
† Those authors were contributed equally.

## Experimental Procedures

*Synthesis of boronic acid ( BA )-linker((3-(2-bromoacetamido)phenyl)boronic acid)*

Synthesis of 3-amino phenyl boronic ester (2)

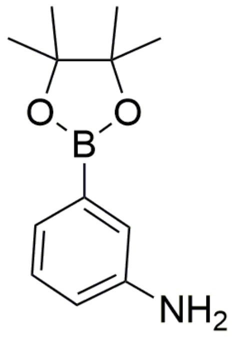


(2)

A mixture of 3-bromoaniline (7.90 g, 45.9 mmol), 4,4,4′,4′,5,5,5′,5′-octamethyl-2,2′-bi(1,3,2-dioxaborolane) (12.8 g, 50.5 mmol), and potassium acetate (9.00 g, 91.80 mmol) in 1,4-dioxane (100.00 mL) was purged with nitrogen for 10 min. The mixture was then treated with PdCl2(dppf) dichloromethane adduct (0.83 g, 4.59 mmol) and bubbled with nitrogen for an additional 5 min. The mixture was heated at reflux for 12 h and then cooled to room temperature and filtered through celite. The pad was washed with ethyl acetate and the filtrate was concentrated under reduced pressure and purified by silica gel chromatography (10–25% ethyl acetate in hexanes) to afford 3-amino phenyl boronic ester (**2**) as a white solid (9.1g, 91.0%). 1H NMR (500 MHz, $CDCl_3$): δ 7.14 (m, 2H), 7.11 (d, 1H, *J*=2.0 Hz), 6.78-6.76 (m, 1H), 3.61 (br, 2H) and 1.32 (s, 12H). 13C NMR (125 MHz, $CDCl_3$): δ =145.95, 128.96, 125.20, 121.34, 118.21, 83.92 and 25.06. HRMS (ESI) *m/z* calcd: $C_{12}H_{18}BNO_2$, 220.1503 [M]; found: $C_{12}H_{18}BNO_2H$, 220.1503 $[M+H]^+$ [1].

Synthesis of ((3-(2-bromoacetamido)phenyl)boronic ester) (3)

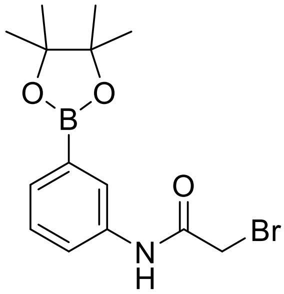

(3)

To a stirred solution of compound **2** (4.00 g, 18.3 mmol) in dichloromethane (100.00 mL) was added $K_2CO_3$ (8.87 g, 64.2 mmol) followed by the dropwise addition of bromoacetyl bromide (3.2 mL, 36.7 mmol) in dichloromethane (50.0 mL) at 0°C for 30 min. The reaction mixture was stirred at room temperature for 5h. The solid was filtered off and the solution was washed with water and dried to get a yellow solid. It was further recrystallized by hexane to get bromo-acetamido phenyl boronic ester (3) as a pale yellow solid. (5.00g, 80%).1H NMR (500 MHz, CDCl3): δ 8.39 (s, 1H), 7.91 (d, 1H, J=7.5Hz), 7.71 (s, 1H), 7.61(d, 1H, J=7.5Hz), 7.38 (t, 1H, J=8.0, 7.5Hz), 4.02 (s, 2H) and 1.35 (s, 12H). 13C NMR (125 MHz, CDCl3): δ = 163.77, 136.56, 131.47, 128.69, 126.06, 123.19, 84.07, 29.56 and 24.89. HRMS (ESI) m/z calcd: $C_{14}H_{19}BBrNO_3$, 339.0641 [M]; found: $C_{14}H_{19}BBrNO_3H$ 340.0720 $[M+H]^+$ [2].

Synthesis of ((3-(2-bromoacetamido)phenyl)boronic acid) (4)

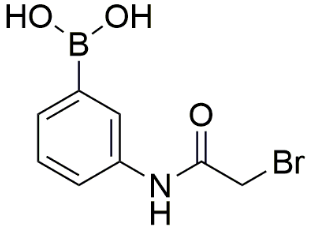

(4)

The compound **3** (5.0 g, 14.7 mmol) was dissolved in mixture of solvents (THF: $H_2O$, 40 mL: 10 mL). NaIO4 was added (9.4 g, 44.1 mmol) and stirred at room temperature for 1h. 2N HCl aqueous solution (4.0 mL) was added and the solution was further stirred at room temperature for 4 h. The reaction mixture was diluted with THF and the solid was filtered off. The solvent was distilled off and the residue was recrystallized by dichloromethane to get bromoacetamido)phenyl)boronic acid (4) as a pale yellow solid. 1H NMR (500 MHz, $CH_3OD$): δ 7. 83 (s, 1H), 7.63 (d, 1H, *J*=7.0Hz), 7.52 (s, 1H), 7.30 (t, 1H, *J*=7.5Hz) and 3.97(s, 2H). 13C NMR (125 MHz, $CH_3OD$): δ = 166.30, 137.23, 129.93, 127.75, 125.43, 122.01 and. 28.38. HRMS (ESI) *m*/*z* calcd: $C_8H_9BBrNO_3$, 256.9859 [M]; found: $C_8H_9BBrNO_3H$ $[M+H]^+$ 257.9943.

*Complexation of boronic acid ( BA )-linker with Gd (III)(Gd-DO3A-Am-PBA)*

Synthesis of DO3(*t*Bu)$_3$-HBr (6)

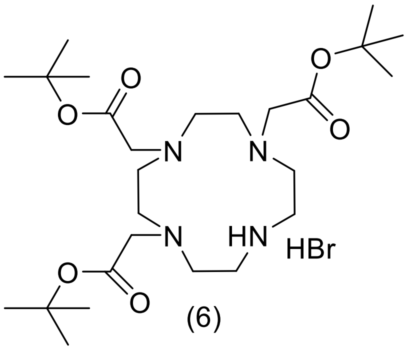


(6)

A solution of cyclen (5.0 g, 29.0 mmol) and anhydrous sodium acetate (7.37 g, 89.9 mmol) in DMAc (100 mL) was cooled to 0˚C before the addition of tert-butyl bromoacetate (16.98 g, 87.06 mmol) in DMAc (50 mL) dropwise. The resulting solution was stirred at 8 ˚C for 60 h. After that, the reaction mixture was poured into a solution of KBr (10.0 g) in 200 mL water and Saturated $NaHCO_3$ solution was added to the solution to precipitate the compound. The precipitate was filtered and washed with water, ether and lypholized to get DO3(*t*Bu)$_3$-HBr (6) as a white solid (21.5g, 60%). 1H NMR (500 MHz, $CDCl_3$): δ3.25-2.74 (m, 22H), 1.34 (s, 18H) and 1.33 (s, 9H). 13C NMR (125 MHz, $CDCl_3$): δ 170.46 169.57, 81.72, 81.54, 58.04, 51.21, 49.07, 47.43, 28.16 and 28.15 HRMS (ESI) *m*/*z* calcd: $C_{25}H_{51}N_4O_6$, 515.3803 [M]; found: $C_{25}H_{51}N_4O_6H$ 515.3803 $[M+H]^+$ [3].

1.2.2. Synthesis of DO3(*t*Bu)$_3$-Am-PBA (7)

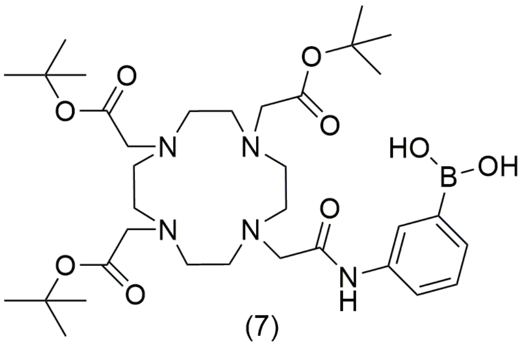


(7)

Compound **6** (3.20 g, 5.37 mmol) and compound **4** (1.52 g, 5.90 mmol) were dissolved in acetonitrile: ethanol (2:1, 90 mL and $K_2CO_3$ (1.48 g, 10.74 mmol) mixture. The solution was stirred at ambient temperature for 48 h, solid was filtered and washed with excess of ethanol. The solvent was removed under reduced pressure and the crude yellow solid was purified by column chromatography (0% to 25% methanol in DCM) to obtain DO3(*t*Bu)$_3$-Am-PBA (7) as a pale yellow solid (1.85g, 50 %). 1H NMR (500 MHz, $CDCl_3$): δ 10.11 (s, 1H), 8.04 (d, 1H, *J*=8.1Hz), 7.78 (s, 1H), 7.52 (d, 1H, *J*=7.3Hz), 7.09 (t, 1H, *J*=7.0, 7.7Hz), 3.44-2.43 (m, 24H), 1.39 (s, 18H) and 1.33 (s, 9H). 13C NMR (125 MHz, $CDCl_3$): δ 172.33, 170.95, 138.16, 129.76, 127.38, 125.70, 122.52, 81.86, 56.79, 27.94, 27.93. HRMS (ESI) *m*/*z* calcd: $C_{34}H_{58}BN_5O_9$, 691.4328 [M]; found: $C_{34}H_{58}BN_5O_9H$ 692.4377 $[M+H]^+$ [3]

1.2.3. Synthesis of DO3A-Am-PBA (8)

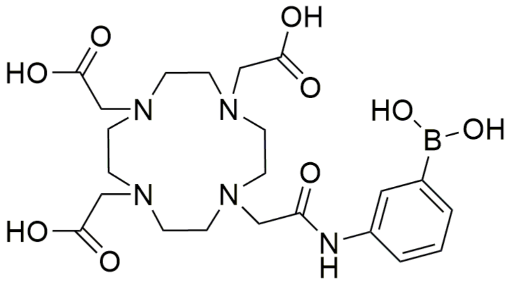


(8)

Compound **6** (1.5 g, 2.17 mmol) was treated with trifluoroacetic acid/dichloromethane (1/2 v/v, 10 mL) and stirred for 12 h at room temperature. The solvent was removed under reduced pressure and the residue was purified by ion exchange resin to get DO3A-Am-PBA(8) as white solid (0.67g, 59.0%).1H NMR (600 MHz, $D_2O$): δ 7.77 (s, 1H), 7.48-7.46 (m, 2H,), 7.41 (t, 1H, *J*=7.58. 7.5Hz), 3.77 (s, 4H), 3.61 (s, 2H), 3.50 (s, 4H), 3.36 (s, 6H), 3.10-2.95 (m, 8H). 13C NMR (150 MHz, $D_2O$): δ= 174.38, 169.99, 136.55, 130.26, 128.67, 126.36, 123.83, 56.42, 55.53, 53.27, 51.37, 50.93, 48.31 and 48.02. HRMS (ESI) *m*/*z* calcd: $C_{22}H_{31}BN_5O_9$, 523.2450 [M]; found: $C_{22}H_{30}BN_5O_9$ $[M -H]^-$ 522.2340 [4].

1.2.4. Synthesis of Gd-DO3A-Am-PBA (9)

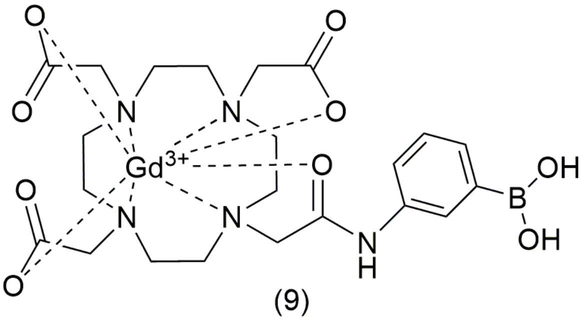


(9)

Compound **8** (0.465 g, 0.88 mmol) and $GdCl_3.6H_2O$ (0.33 g, 0.88 mmol) were dissolved in $H_2O$ (15 mL) and the pH was adjusted to 5.5 using 1 M NaOH. The solution was stirred at 50°C for 18 h and the pH was adjusted to 7. The insoluble material was filtered off and the solvent was dried at room temperature to get white residue which was lyophilized to get Gd-DO3A-Am-PBA (9) as white solid. The absence of free Gd was confirmed by xylenol orange test. HRMS (ESI) *m*/*z* calcd: $C_{22}H_{31}BGdN_5O_9$, 679.1439 [M]; found: $C_{22}H_{31}BGdN_5O_9H$ 679.1545 $[M +H]^+$, 717.1102 $[M +K]^+$, HPLC purity 97.74%.

*Cell melanogenesis dermination*

Melanogenic and non melanogenic cells were grown until they reach 70-80% confluency. Rate of melanogenesis in melanoma and non-melanoma cells were determined by absorbance measurement of the cell lysates. At every successive passage, cells were washed with PBS, trypsinized, counted and collected in 1.5 mL microcentrifuge tubes [5]. The pelleted cells were lysed in 100ul of 1 N NaOH containing 10% DMSO and the melanin content was determined by measuring the absorbance at 490 nm in a microplate reader (SpectraMax 190; Molecular Devices). Bright field images of the cells obtained by culturing

at different pH and medium are shown in Figure S1. Thus obtained non melanoma cells were used as reference cells for *in vitro* labeling experiments.

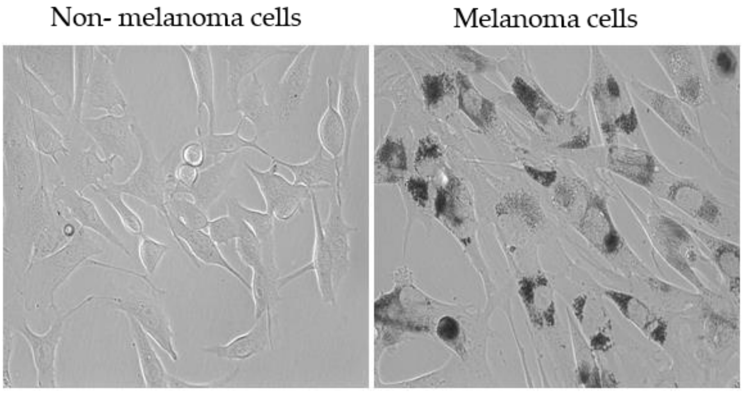


**Figure S1.** 40X Brightfield images of non-melanoma and melanoma cells under light microscope.

*In vitro MRI experiments*

The phantom relaxometry experiments were performed on a 7-T spectrometer (PharmaScan 70/16, Bruker, Germany). A birdcage coil with a 72 mm inner diameter was used for radio frequency excitation and signal reception. T1 maps were generated using T1map_RARE with variable TRs (8000、4000、2000、800、600、300、150、60 ms) and field of view (FOV)=7×7 cm; echo time (TE) =7 ms; number of excitations (NEX)=2; matrix size=256×256; slice-thickness=1.5 mm. T2 maps were generated using a multi spin-echo sequence with field of view (FOV)=7×7 cm; TR=5500 ms; TEs=8, 16 to 112 ms with increments of 8 ms; NEX=2; resolution=256×256; slice-thickness=1.5 mm. T1 and T2 maps were generated by fitting the signal intensity data of the various TRs and TEs, respectively, for the exponential T1 and T2 curves on a voxel by voxel basis using MRVision (MRVision Co., Menlo Park, CA, U.S.A.). The two parameter fits to mono-exponential models were used. R1 and R2 were calculated as the inverse of T1 and T2, respectively. During acquisition, the tubes were positioned parallel to the main magnetic field (B0) along the z axis in the magnet.

*In vivo MRI*

All images were acquired with a 7-T spectrometer (PharmaScan 70/16, Bruker, Germany scanner. The scanner used a 38-mm volume coil for signal transmission and reception. T2-weighted imaging (T2WI) was used to acquire anatomical images. Detection was done using a Rapid Acquisition with Relaxation Enhancement (RARE) sequence with a TR of 4000 ms, TE of 50 ms, 4 averages, FOV= 4 × 4 cm, slice thickness of 0.8 mm, 28 slices, and acquisition matrix = 256×256. pre- and post-T1-weighted im mages (T1WI) were acquired using RARE spin echo sequence with a TR of 1000 ms, TE of 9 ms, 4 averages, FOV = 4 × 4 cm, slice thickness of 0.8 mm, and acquisition

matrix = 256×256 after the intravenous injection of 0.1 mmol/kg of Gd-DO3A-Am-PBA via tail vein. Control group mice experiments were performed using the same scanning protocol of T1WI and T2WI, and contrast agent intravenous injection of 0.1 mmol/kg of Gadovist via a tail vein.

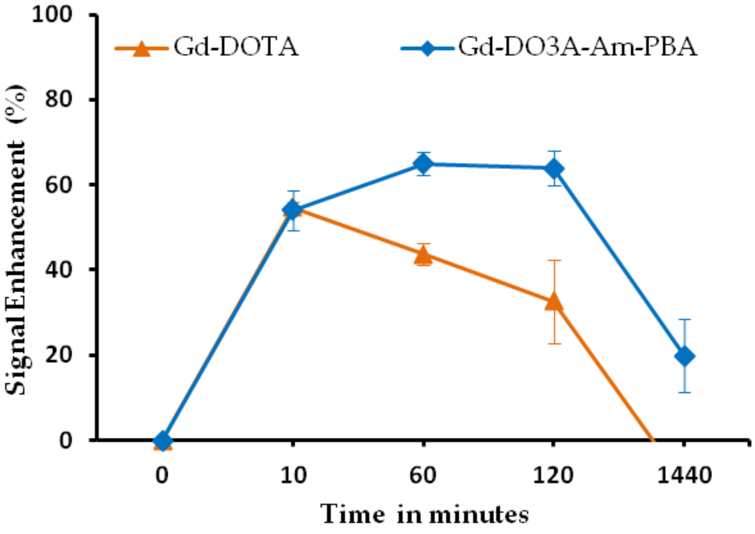


**Figure S2.** Quantification and comparison of SNR in the tumor region measured after the intratumor injection of Gd-DOTA and Gd-DO3A–Am-PBA are shown. Error bars represent mean ± standard error values. SNR : signal- to- noise ratio.

## References


1. Xu, D.; Sun, D.; Wang, W.; Peng, X.; Zhan, Z.; Ji, Y.; Shen, Y.; Geng, M.; Ai, J.; Duan, W. Discovery of pyrrolo[2,3-d]pyrimidine derivatives as potent Axl inhibitors: Design, synthesis and biological evaluation. *Eur. J. Med. Chem.* **2021**, *220*, 113497.
2. Lee, S. W.; Lee, S. Y.; Lee, S. H. Self-assembly of pyrene boronic acid-based chemodosimeters for highly efficient mercury(II) ion detection. *Tetrahedron Lett.* **2019**, *60*, 151048.
3. Sun, C.; Lin, H.; Gong, X.; Yang, Z.; Mo, Y.; Chen, X.; Gao, J. DOTA-Branched Organic Frameworks as Giant and Potent Metal Chelators. *J. Am. Chem. Soc.* **2020**, *142*, 198–206.
4. Geninatti Crich, S.; Alberti, D.; Szabo, I.; Aime, S.; Djanashvili, K. MRI Visualization of Melanoma Cells by Targeting Overexpressed Sialic Acid with a GdIII-dota-en-pba Imaging Reporter. *Angew. Chem. Int. Ed.* **2013**, *52*, 1161–1164.
5. Szabó, I.; Crich, S. G.; Alberti, D.; Kálmán, F. K.; Aime, S. Mn loaded apoferritin as an MRI sensor of melanin formation in melanoma cells. *Chem. Commun.* **2012**, *48*, 2436–2438.
